# Interaction of sex and insulin resistance with cognitive impairment in community-dwelling middle-aged and older adults

**DOI:** 10.3389/fnagi.2026.1889416

**Published:** 2026-10-16

**Authors:** Jung Sook Kim, Byung Chul Chun, Joong-Yeon Lim

**Affiliations:** 1 Division of Population Health Research, Department of Precision Medicine, Korea National Institute of Health, Cheongju-si, Chungcheongbuk-do, Republic of Korea; 2 Department of Preventive Medicine, Korea University College of Medicine, Seoul, Republic of Korea

**Keywords:** aging, cognitive impairment, insulin resistance, interaction, sex factors

## Abstract

**Introduction:**

Cognitive impairment is a major global health challenge. However, whether sex and age interactively varies the association between insulin resistance and cognitive impairment remains unclear. This cross-sectional study aimed to evaluate the association between insulin resistance and cognitive impairment and identify sex- and age-specific interactions in community-dwelling adults.

**Methods:**

The study involved 3,898 participants (aged 54–86 years) from the Ansan-Ansung cohort of the Korean Genome and Epidemiology Study (2017–2018). Insulin resistance was assessed via Homeostatic Model Assessment for Insulin Resistance (HOMA-IR), with participants dichotomized by the median (1.97). Cognitive impairment was defined as a score of ≤23 on the Korean Mini-Mental State Examination score. Multivariable logistic regression was applied to examine the interaction and stratified effects of sex and age (≥65 vs. <65 years) on the insulin resistance–cognitive impairment association.

**Results:**

Among 3,898 participants (mean age 66.6 years; 55.5% women), 635 (16.3%) had cognitive impairment. A significant interaction was observed between sex and HOMA-IR (*p* = 0.04), whereas the interaction for age was not significant (*p* = 0.17). In stratified analyses, higher HOMA-IR was significantly associated with cognitive impairment exclusively in women aged ≥65 years (OR 1.37; 95% CI 1.02–1.84; *p* = 0.04).

**Conclusion:**

Higher insulin resistance is a significant modifiable factor for cognitive impairment, specifically in women aged ≥65 years. These exploratory findings suggest potential sex-related differences in the metabolic-cognitive association, underscoring the importance of sex-specific metabolic management starting from midlife to help preserve cognitive health in later life.

## 1 Introduction

Cognitive impairment and metabolic abnormalities have emerged as major public health challenges globally, largely driven by population aging. Aging is the primary risk factor for neurodegenerative diseases, including Alzheimer’s disease (AD) (Hou et al., 2019). AD, the most prevalent form of dementia, imposes a substantial socioeconomic burden because of the lack of effective treatments (Ezkurdia et al., 2023). It progresses along a continuum from the preclinical stage to mild cognitive impairment (MCI) and ultimately to overt dementia (Porsteinsson et al., 2021). Cognitive changes may begin up to 20 years before a clinical diagnosis of MCI (Caselli et al., 2020). Identifying modifiable risk factors during the preclinical stage is crucial for early detection and preventive interventions.

Insulin regulates glucose metabolism in peripheral tissues and exerts critical effects in the brain, influencing neuronal metabolism, maintaining glucose homeostasis, and supporting cognitive function (Cui et al., 2022). Brain insulin signaling is involved in both physiological homeostasis and neuropathological processes, including cognitive decline and AD (Milstein and Ferris, 2021). Moreover, reduced brain insulin action (or brain insulin resistance [IR]) has been linked to metabolic disturbances such as obesity and type 2 diabetes, as well as to aging and AD, highlighting a potential association between metabolic and cognitive health (Agrawal et al., 2021). Furthermore, brain IR is a prominent feature of the aging brain, even in the absence of systemic diabetes, and may directly contribute to the progression of cognitive impairment (Rhea et al., 2024).

Sex-specific factors may further modulate this metabolic–cognitive link. Estrogen influences hippocampal learning and memory, and its postmenopausal reduction is associated with an increased risk of AD (Singh and Paramanik, 2022). Consistent with this biological vulnerability, a recent meta-analysis of longitudinal tau positron emission tomography studies reported faster tau accumulation in women with high baseline amyloid-β pathology than in men (Coughlan et al., 2025). These findings suggest that sex-specific vulnerability in AD-related pathology emerges early in the disease continuum. However, whether IR contributes to these sex differences in cognitive impairment remains unclear.

Although previous studies (Geroldi et al., 2005; Ekblad et al., 2015; Kong et al., 2018; Gruber et al., 2024) have reported associations between IR and cognitive decline, the clinical implications of these glycemic markers differ conceptually. HOMA-IR serves as a proximal, mechanistically informative predictor of cognitive decline, reflecting insulin resistance that underlies early metabolic dysfunction. In contrast, metrics derived from continuous glucose monitoring (CGM) are more relevant in older adults with established diabetes, a clinical context where profound glycemic variability and hypoglycemia directly contribute to neurotoxicity (Gruber et al., 2024). Therefore, in this study, we aimed to investigate the cross-sectional association between IR and cognitive impairment in community-dwelling Korean adults aged 54–86 years and explore the potential interactions of sex and age with insulin resistance. Our findings may inform targeted prevention strategies for cognitive impairment in aging populations.

## 2 Methods

### 2.1 Study participants

The Korean Genome and Epidemiology Study (KoGES) is a large-scale cohort project launched in 2001 by the National Institute of Health, the Korea Disease Control and Prevention Agency, to identify risk factors for common chronic diseases in Koreans (Kim and Han, 2017). The community-based Ansan-Ansung cohort was established in 2001–2002 as part of the KoGES, enrolling 10,030 adults aged 40–69 years. This study employed a cross-sectional design using data from the 9th-wave (2017–2018) survey of the ongoing KoGES Ansan-Ansung cohort. This specific wave was selected because it represents the first major follow-up cycle of the specialized ‘Advanced Aging Study’ (baseline established in 2011–2014) where both multi-domain cognitive scores and fasting insulin/glucose levels were concurrently completed. A cross-sectional framework was adopted as a necessary initial step to establish the baseline sex- and age-specific interaction profiles and stratified associations within this newly matured aging framework, which serves as a mandatory methodological prerequisite prior to examining long-term prospective longitudinal trajectories. Of the 6,157 participants, 3,898 (aged 54–86 years) with complete cognitive and clinical assessments were analyzed (Figure 1).

**Figure 1:**
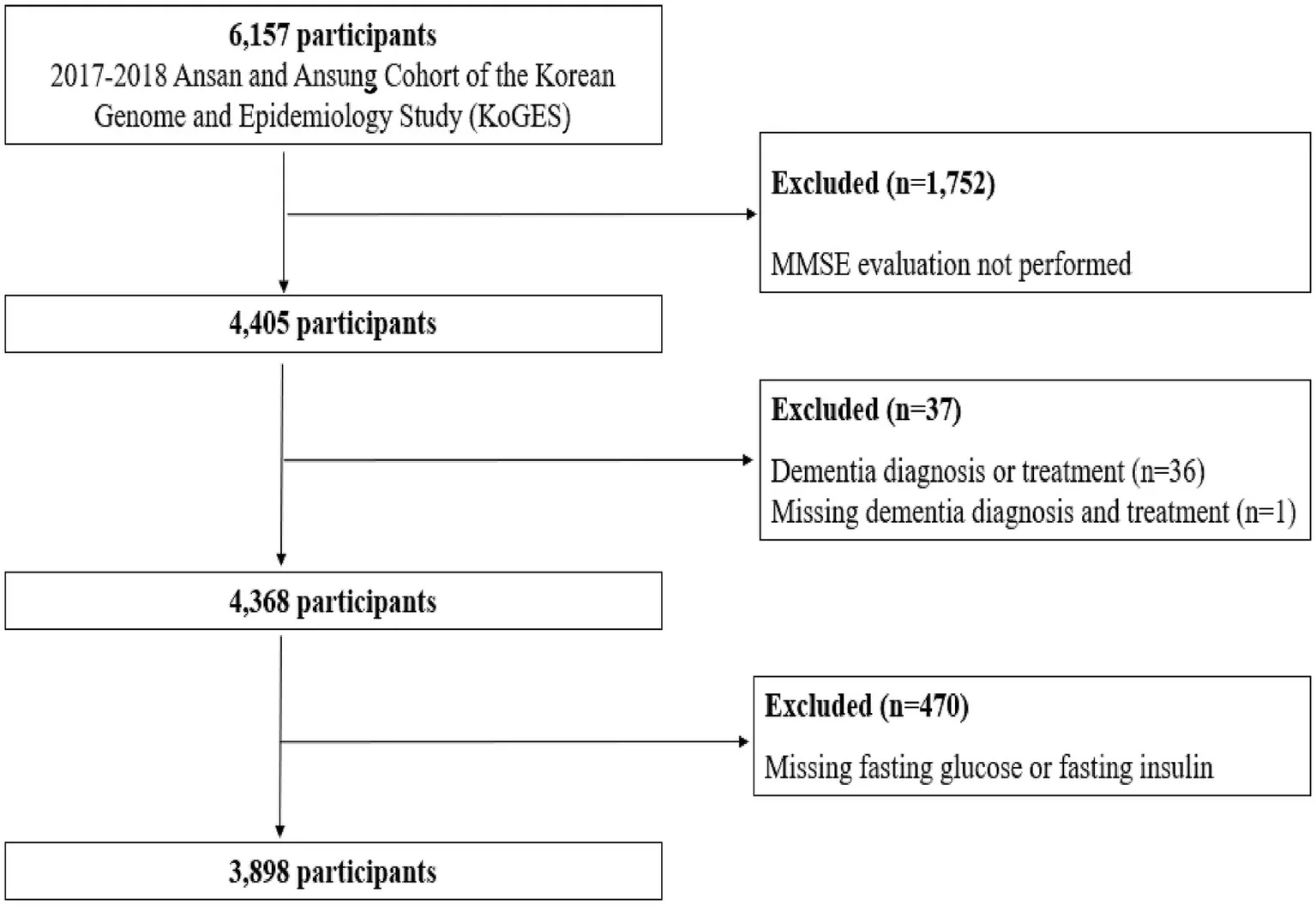
Flowchart of study participant selection.

Participants were selected based on the following exclusion criteria: (i) absence of Korean version of the Mini-Mental State Examination (K-MMSE) scores; (ii) a prior diagnosis of or treatment for dementia, including missing information on dementia status; or (iii) missing fasting glucose or insulin data required for the HOMA-IR calculation (Figure 1). This study adhered to the Strengthening the Reporting of Observational Studies in Epidemiology reporting guidelines.

The study protocol was approved by the Institutional Review Board of the Korea National Institute of Health, the Korea Disease Control and Prevention Agency (Approval No. KDCA-2025-07-02-P-01). All participants provided written informed consent before participation.

### 2.2 Cognitive impairment

Cognitive function was assessed using the K-MMSE, a validated tool with 0.70–0.83 sensitivity for dementia (Kang et al., 1997). It comprises seven domains (total score 0–30): orientation in time, orientation in place, memory registration, attention and calculation, memory recall, oral and written language ability, and visuospatial function (Supplementary Table 1), with higher scores indicating better cognitive function.

A total of 3,898 participants completed the K-MMSE. Cognitive function was initially categorized into severe cognitive impairment (SCI; score ≤ 17, *n* = 97), MCI (18–23, *n* = 538), and normal cognitive function (≥24, *n* = 3,263) (Kang et al., 1997; Euna Oh et al., 2010; Kim et al., 2024). Given the small sample size in the SCI group, participants with SCI and MCI were combined into a single “cognitive impairment” group (score ≤ 23; *n* = 635, 16.3%), which was designated as the dependent variable in this study. The remaining participants (≥24, *n* = 3,263, 83.7%) were defined as the “normal cognition” group.

### 2.3 Insulin resistance

As the independent variable of interest, insulin resistance (IR) was calculated using the HOMA-IR formula: (fasting insulin [μU/mL] × fasting glucose [mg/dL])/405 (Matthews et al., 1985). Participants were dichotomized into low and high HOMA-IR groups based on a median value of 1.97 (Supplementary Table 2). This threshold was selected because HOMA-IR values were non-normally distributed (Kolmogorov–Smirnov test, *p* = 0.010), minimizing the influence of extreme outliers. Furthermore, this threshold aligns with prior research that has used a HOMA-IR cutoff of 2.0 to define IR in relation to cognitive function (Gruber et al., 2024) and follows established methodological approaches for median-based categorization (Song et al., 2007).

### 2.4 Covariates

Potential confounding covariates were evaluated, including sociodemographic factors (sex, age, and education), lifestyle factors (smoking, alcohol, and exercise), anthropometric measures (body mass index, blood pressure, waist circumference, and muscle mass), and biochemical markers (glucose, insulin, lipids, vitamin D, and high-sensitivity C-reactive protein). Age was dichotomized at 65 years (median; Lancet late-life definition, Livingston et al., 2024). Given the significant influence of educational attainment on cognitive performance, educational level was rigorously adjusted for as a key covariate in all multivariable regression models to minimize potential confounding bias on K-MMSE scores. Depressive symptoms were defined as a score of ≥6 on the Korean Short Form Geriatric Depression Scale (Lee et al., 2013; Kong et al., 2018). This factor is linked to lower cognitive scores and increased risks of MCI and AD (Mehta et al., 2022). All clinical and cognitive assessments followed the standardized KoGES protocols.

### 2.5 Statistical analysis

Continuous variables are presented as mean ± standard deviation or median (interquartile range), and categorical variables are presented as frequencies and percentages. MMSE domain scores were scaled to 0–100, and the mean scaled scores were calculated. Group comparisons for baseline characteristics were performed using Student’s *t*-test and the *χ*^2^ test. To evaluate the association between the independent variable (HOMA-IR group) and the dependent variable (cognitive impairment, defined as an MMSE score ≤ 23), multivariable logistic regression analysis was conducted using cognitive impairment (K-MMSE score ≤ 23) as the outcome (1) and normal cognition (K-MMSE score ≥ 24) as the reference (0).

Covariates with *p* < 0.20 in univariate analyses were included in the multivariable-adjusted models. To ensure model stability and methodological rigor, we employed a three-stage variable selection process: (1) Univariable screening: To identify potential confounders while balancing variable inclusion and model parsimony, we performed univariable analysis with a liberal threshold of *p* < 0.20. This strategy prevents the premature exclusion of relevant covariates, thereby minimizing the risk of residual confounding. Specifically, variables meeting this threshold (such as HOMA-IR, sex, age, education, alcohol consumption, smoking, physical inactivity, blood pressure [SBP, DBP], waist circumference, muscle mass, total cholesterol, and depression) were advanced as candidates, while those failing to meet it (such as BMI, triglycerides, HDL-cholesterol, Vitamin D, and hsCRP) were excluded. (2) Model construction: Candidate variables identified from the screening stage were entered into multivariable logistic regression models using the simultaneous entry method. The final model included: continuous variables (HOMA-IR, systolic/diastolic blood pressure, waist circumference, muscle mass, total cholesterol, and depression) and categorical variables (age categorized by 65 years, sex, education, alcohol consumption, smoking, and physical inactivity). For the age- and sex-stratified subgroups, the respective stratification variables (age categorized by 65 years and/or sex) were naturally excluded from those specific subgroup models. (3) Diagnostic stage: We assessed multicollinearity using the variance inflation factor (VIF). To maintain scientific integrity, fasting insulin (VIF = 12.14) and fasting glucose (VIF = 2.88, excluded due to mathematical redundancy with HOMA-IR [HOMA-IR VIF = 15.06]) were removed. Following the exclusion of fasting insulin and fasting glucose, all retained variables in the final multivariable regression model were evaluated to ensure the absence of multicollinearity (all VIFs ranging from 1.05 to 4.80, all < 5).

Analyses followed a staged approach, involving a crude model, a minimally adjusted model (age and sex), and interaction models. To justify the stratified approach, interaction terms for (sex [women] × HOMA-IR [≥1.97]) and (age [≥65 years] × HOMA-IR [≥1.97]) were tested in the total population. To evaluate the robustness of the associations, age was treated as a continuous variable in the sex-stratified analysis of the total population, men, and women. Subsequently, age was dichotomized at 65 years for subgroup analyses of the total population and four age-sex combined groups.

Model calibration and discrimination were assessed using the Hosmer–Lemeshow test and area under the receiver operating characteristic curve (AUC). Adequate model calibration (*P_H_*–*_L_* > 0.05) was primarily demonstrated in the female-specific and women aged ≥65 years models, whereas the total population model showed sub-optimal fit. Finally, MMSE domain scores were compared between the HOMA-IR groups within each sex using Student’s *t-*test (Supplementary Table 3). For subgroup analyses of the Mini-Mental State Examination (MMSE) domains, we applied the Bonferroni correction to account for multiple comparisons. Adjusted *p*-values were obtained by multiplying the raw *p*-values by seven, reflecting the number of MMSE subdomains evaluated. Any adjusted *p*-values exceeding 1.000 were reported as 1.000. All analyses were performed using SAS version 9.4 (SAS Institute Inc., Cary, NC, USA). Two sided *p* < 0.05 was considered statistically significant.

## 3 Results

### 3.1 Participant characteristics and prevalence of cognitive impairment

Of the 6,157 individuals followed up in the 9th wave, 1,752 were excluded for not undergoing the K-MMSE, 37 were excluded due to dementia (36 previously diagnosed/treated and one with missing data), and 470 were excluded for missing fasting glucose or insulin data (required for HOMA-IR calculation). Finally, the study population included 3,898 individuals (Figure 1).

Among the 3,898 participants (mean age, 66.6 years), 16.3% had cognitive impairment (Table 1). Although high HOMA-IR (HHG) prevalence did not differ by sex (*p* = 0.21; Supplementary Table 4), women and older adults (≥65 years) exhibited a significantly higher prevalence of cognitive impairment (both *p* < 0.0001; Supplementary Table 5). Higher depressive symptoms were associated with cognitive impairment (*p* < 0.0001; Supplementary Table 5), but not with HHG status (Supplementary Table 6). The educational attainment of the study population was diverse; 57.9% of participants had completed middle school or less, while 42.1% had completed high school or higher. This variability was accounted for by adjusting for educational level as a key covariate in all multivariable regression models.

**Table 1:** Clinical characteristics and cognitive function of the study participants.

Variable	Category	*N*	*n* (%)	Mean ± SD
Sex	Men	3,898	1,735 (44.5)	
	Women		2,163 (55.5)	
Age, year (continuous)	—	3,898	—	66.6 ± 8.2
Age, year (categorical)	<65	3,898	1,939 (49.7)	
	≥65		1,959 (50.3)	
Education	≤Middle school	3,868	2,239 (57.9)	
	≥High school		1,629 (42.1)	
Alcohol consumption	Never drinker	3,897	2,270 (58.3)	
	Past drinker		251 (6.4)	
	Current drinker		1,376 (35.3)	
Smoking	Never smoker	3,898	2,616 (67.1)	
	Past smoker		896 (23.0)	
	Current smoker		386 (9.9)	
Regular exercise	No	3,898	2,638 (67.7)	
	Yes		1,260 (32.3)	
BMI, kg/m^2^	—	3,834	—	24.7 ± 3.2
SBP, mm Hg	—	3,881	—	125.1 ± 17.2
DBP, mm Hg	—	3,881	—	78.1 ± 9.9
WC, cm	—	3,898	—	86.0 ± 8.7
Muscle mass, kg	—	3,834	—	41.0 ± 8.2
TC, mg/dL	—	3,897	—	186.0 ± 37.3
TG, mg/dL	—	3,895	—	136.3 ± 86.7
HDL-C, mg/dL	—	3,897	—	45.9 ± 11.8
Vitamin D, ng/mL	—	3,673	—	21.0 ± 10.2
hsCRP, mg/L	—	3,897	—	1.8 ± 5.3
Fasting glucose, mg/dL	—	3,898	—	99.6 ± 26.1
Fasting insulin, μIU/mL	—	3,898	—	9.5 ± 5.2
HOMA-IR (continuous)	—	3,898	—	2.4 ± 1.8
HOMA-IR (categorical)	≥1.97 (high group)	3,898	1,956 (50.2)	
	<1.97 (low group)		1,942 (49.8)	
GDS score (continuous)	—	3,891	—	2.1 ± 3.1
GDS score (categorical)	≥6 (depressive symptoms)	3,891	487 (12.5)	
	<6 (normal)		3,404 (87.5)	
MMSE score (continuous)	—	3,898	—	26.5 ± 3.4
MMSE score (categorical)	≤23 (cognitive impairment)	3,898	635 (16.3)	
	≥24 (normal cognition)		3,263 (83.7)	

The HOMA-IR was dichotomized at the median value (≥1.97 = high group; <1.97 = low group). The GDS score was categorized using a cutoff of ≥6 to indicate depressive symptoms. An MMSE score of ≤23 was used to define cognitive impairment. Educational level was categorized as ≤middle school or ≥high school. Alcohol consumption and smoking status were classified as never, past, or current users. Regular exercise was coded as yes or no. Continuous variables are presented as mean ± standard deviation, and categorical variables are expressed as number (%).

BMI, body mass index; DBP, diastolic blood pressure; GDS, Geriatric Depression Scale; HDL-C, high-density lipoprotein cholesterol; HOMA-IR, homeostatic model assessment of insulin resistance; hsCRP, high-sensitivity C-reactive protein; MMSE, Mini-Mental State Examination; SBP, systolic blood pressure; TC, total cholesterol; TG, triglycerides; WC, waist circumference.

### 3.2 Sex differences in the association between IR and cognitive impairment

We examined the association between HHG and cognitive impairment using crude, minimally adjusted, and interaction models (Table 2). In the crude analysis, HHG was significantly associated with an increased odds of cognitive impairment (odds ratio [OR], 1.36; 95% confidence interval [CI], 1.14–1.61). However, this association was no longer significant after adjusting for sex and age (OR, 1.19; 95% CI, 0.99–1.43). Interaction analyses revealed a significant interaction between sex and HHG (*P* for interaction = 0.04, though no longer significant after Bonferroni correction), whereas no interaction was observed for the age group (*P* for interaction = 0.17).

**Table 2:** Association between insulin resistance and cognitive impairment: crude, minimally adjusted, and interaction models.

Variable	Crude model MMSE decline OR (95% CI)	Minimally adjusted model MMSE decline OR (95% CI)	Sex interaction model MMSE decline OR (95% CI)	Age interaction model MMSE decline OR (95% CI)
HOMA-IR (≥1.97)	1.36 (1.14, 1.61)^**^	1.19 (0.99, 1.43)	1.14 (0.90, 1.43)	1.15 (0.91, 1.45)
Sex (women)	—	2.67 (2.19, 3.26)^***^	0.41 (0.26, 0.66)^**^	0.47 (0.30, 0.74)^**^
Age (≥65 years)	—	7.15 (5.68, 8.99)^***^	2.90 (2.20, 3.81)^***^	2.62 (1.91, 3.60)^***^
Sex (women) × HOMA-IR	—	—	1.06 (1.002, 1.124)^†^	—
Age (≥65 years) × HOMA-IR	—	—	—	1.04 (0.98, 1.11)
Education (≤middle school)	—	—	4.24 (3.07, 5.87)^***^	4.24 (3.07, 5.87)^***^
Alcohol consumption (ever)	—	—	1.12 (0.86, 1.46)	1.11 (0.85, 1.45)
Smoking (ever)	—	—	0.76 (0.52, 1.11)	0.76 (0.52, 1.11)
Regular exercise (no)	—	—	1.53 (1.19, 1.97)^**^	1.53 (1.19, 1.98)^**^
SBP, mm Hg	—	—	1.01 (1.00, 1.02)^**^	1.01 (1.00, 1.02)^**^
DBP, mm Hg	—	—	0.99 (0.98, 1.01)	0.99 (0.98, 1.01)
WC, cm	—	—	1.02 (1.00, 1.03)^*^	1.02 (1.00, 1.03)^*^
Muscle mass, kg	—	—	0.90 (0.87, 0.92)^***^	0.90 (0.87, 0.92)^***^
TC, mg/dL	—	—	1.00 (1.00, 1.00)	1.00 (1.00, 1.00)
GDS score	—	—	1.08 (1.05, 1.11)^***^	1.08 (1.05, 1.11)^***^

HOMA-IR was dichotomized at the median (≥1.97 = 1). Sex was coded as women = 1; age was dichotomized at 65 years (≥65 years = 1). Educational level (≤middle school), alcohol consumption (ever drinker), smoking (ever smoker), and regular exercise (no) were coded as 1; otherwise, 0. Interaction terms include Sex × HOMA-IR and Age × HOMA-IR, using the above coding. All models used logistic regression, with cognitive impairment as the dependent variable. Crude model was adjusted for the HOMA-IR only; minimally adjusted model was additionally adjusted for sex and age; interaction models further included interaction terms and covariates: education, alcohol consumption, smoking, exercise, SBP, DBP, WC, muscle mass, TC, and GDS score. ^*^p < 0.05; ^**^p < 0.01; ^***^p < 0.0001; ^†^P for interaction (Sex × HOMA-IR) = 0.04.

OR, odds ratio; CI, confidence interval; MMSE, Mini-Mental State Examination; HOMA-IR, homeostatic model assessment of insulin resistance; SBP, systolic blood pressure; DBP, diastolic blood pressure; WC, waist circumference; TC, total cholesterol; GDS, Geriatric Depression Scale.

Following the significant sex interaction, sex-stratified analyses were performed after adjusting for continuous age and other covariates (Table 3). In the total population, HHG was associated with cognitive impairment (OR, 1.26; 95% CI, 1.01–1.57). When stratified by sex, this association remained statistically significant only in women (OR, 1.33; 95% CI, 1.01–1.75), but not in men (OR, 1.05; 95% CI, 0.71–1.55). Continuous age was consistently associated with cognitive impairment across the total population (OR, 1.10; 95% CI, 1.08–1.12), men (OR, 1.05; 95% CI, 1.02–1.08), and women (OR, 1.12; 95% CI, 1.10–1.14).

**Table 3:** Multivariable logistic regression analysis of factors associated with cognitive impairment stratified by sex.

Variable	Cognitive impairment, OR (95% CI)		
	Total population	Men	Women
HOMA-IR (categorical)			
<1.97 (low group)	1 (Reference)	1 (Reference)	1 (Reference)
≥1.97 (high group)	1.26 (1.01, 1.57)^*^	1.05 (0.71, 1.55)	1.33 (1.01, 1.75)^*^
Sex (women)	0.83 (0.51, 1.33)	—	—
Age, year (continuous)	1.10 (1.08, 1.12)^***^	1.05 (1.02, 1.08)^**^	1.12 (1.10, 1.14)^***^
Education (≤middle school)	3.83 (2.76, 5.32)^***^	2.48 (1.65, 3.73)^***^	9.51 (4.76, 18.99)^***^
Alcohol consumption (ever)	1.19 (0.91, 1.57)	1.23 (0.81, 1.86)	1.23 (0.85, 1.79)
Smoking (ever)	0.81 (0.55, 1.18)	0.75 (0.50, 1.11)	0.90 (0.29, 2.76)
Regular exercise (no)	1.38 (1.07, 1.79)^*^	1.59 (1.02, 2.51)^*^	1.30 (0.95, 1.79)
SBP, mm Hg	1.00 (1.00, 1.01)	1.01 (0.99, 1.02)	1.00 (0.99, 1.01)
DBP, mm Hg	1.00 (0.99, 1.02)	1.00 (0.97, 1.02)	1.00 (0.99, 1.02)
WC, cm	1.01 (1.00, 1.03)	1.01 (0.98, 1.04)	1.01 (0.99, 1.03)
Muscle mass, kg	0.94 (0.91, 0.96)^***^	0.94 (0.90, 0.98)^**^	0.92 (0.89, 0.96)^**^
TC, mg/dL	1.00 (1.00, 1.00)	1.00 (0.99, 1.00)	1.00 (1.00, 1.00)
GDS score (continuous)	1.07 (1.04, 1.10)^***^	1.07 (1.01, 1.13)^*^	1.07 (1.04, 1.11)^***^

Sex was coded as women = 1 or men = 0. Educational level was categorized as ≤middle school or >middle school. Alcohol consumption and smoking status were coded as ever (yes) or never (no). Regular exercise was coded as no = 1 or yes = 0. The HOMA-IR was dichotomized at the median: <1.97 (low group, reference) and ≥1.97 (high group); Hosmer–Lemeshow *p* = 0.0002 (total population) and *p* = 0.08 (women); area under the curve = 0.84 (total population) and 0.85 (women). ^*^p < 0.05, ^**^p < 0.01, ^***^p < 0.0001.

OR, odds ratio; CI, confidence interval; HOMA-IR, homeostatic model assessment of insulin resistance; SBP, systolic blood pressure (mm Hg); DBP, diastolic blood pressure (mm Hg); WC, waist circumference (cm); muscle mass (kg); TC, total cholesterol (mg/dL).

### 3.3 Identification and characteristics of the high-risk group in subgroup analyses

We conducted subgroup analyses by age and sex to identify the group most affected by IR (Table 4). When age was dichotomized at 65 years in the total population, the association between HHG and cognitive impairment lost significance (OR, 1.23; 95% CI, 0.99–1.52), contrasting with the continuous age model. Hence, the impact of HHG was not uniform across the population but was concentrated within a specific demographic, which may be masked when age is analyzed as a categorical variable in the total sample.

Among the four subgroups, HHG was significantly associated with cognitive impairment, specifically in women aged ≥65 years (OR, 1.37; 95% CI, 1.02–1.84; *p* = 0.04, though no longer significant after Bonferroni correction). Conversely, no significant associations were observed in women aged <65 years or in men of either age group. Within this high-risk subgroup, other significant predictors included low educational attainment (OR, 9.59; 95% CI, 4.11–22.36), physical inactivity (OR, 1.75; 95% CI, 1.22–2.50), and higher depressive symptoms (OR, 1.08; 95% CI, 1.04–1.12) (Table 4).

**Table 4:** Multivariable logistic regression analysis of factors associated with cognitive impairment stratified by age and sex.

Variable		Cognitive impairment, OR (95% CI)			
	Total population	Age <65 years (*n* = 1,939, 49.7%)		Age ≥65 years (*n* = 1,959, 50.3%)	
		Men (*n* = 905, 46.7%)	Women (*n* = 1,034, 53.3%)	Men (*n* = 830, 42.4%)	Women (*n* = 1,129, 57.6%)
HOMA-IR (categorical)					
<1.97 (low group)	1 (reference)	1 (reference)	1 (reference)	1 (reference)	1 (reference)
≥1.97 (high group)	1.23 (0.99, 1.52)^‡^	1.25 (0.60, 2.58)	1.09 (0.58, 2.07)	0.89 (0.56, 1.43)	1.37 (1.02, 1.84)^*^
Sex (women)	0.48 (0.31, 0.75)^**^	—	—	—	—
Age (≥65 years)	2.92 (2.22, 3.85)^***^	—	—	—	—
Education (≤middle school)	4.22 (3.05, 5.83)^***^	3.33 (1.71, 6.49)^**^	12.86 (3.90, 42.46)^***^	2.29 (1.37, 3.81)^**^	9.59 (4.11, 22.36)^***^
Alcohol consumption (ever)	1.12 (0.85, 1.46)	0.68 (0.33, 1.40)	0.99 (0.48, 2.05)	1.62 (0.97, 2.69)	1.15 (0.75, 1.77)
Smoking (ever)	0.76 (0.52, 1.10)	0.54 (0.26, 1.12)	—^†^	0.74 (0.46, 1.20)	0.98 (0.30, 3.25)
Regular exercise (no)	1.54 (1.19, 1.98)^**^	2.18 (1.00, 4.76)	0.89 (0.47, 1.70)	1.48 (0.85, 2.57)	1.75 (1.22, 2.50)**
SBP, mm Hg	1.01 (1.00, 1.02)^**^	1.04 (1.01, 1.07)^*^	1.03 (1.00, 1.05)	1.01 (0.99, 1.02)	1.01 (1.00, 1.02)
DBP, mm Hg	0.99 (0.98, 1.01)	0.96 (0.91, 1.01)	0.96 (0.92, 1.00)	0.99 (0.97, 1.02)	1.00 (0.98, 1.02)
WC, cm	1.02 (1.01, 1.03)^**^	1.00 (0.94, 1.06)	1.03 (0.99, 1.08)	1.03 (0.99, 1.06)	1.02 (1.00, 1.04)
Muscle mass, kg	0.90 (0.87, 0.92)^***^	0.98 (0.91, 1.05)	0.95 (0.86, 1.04)	0.88 (0.84, 0.93)^***^	0.86 (0.83, 0.90)^***^
TC, mg/dL	1.00 (1.00, 1.00)	1.00 (0.99, 1.01)	1.00 (0.99, 1.01)	1.00 (0.99, 1.01)	1.00 (1.00, 1.00)
GDS score (continuous)	1.08 (1.05, 1.11)^***^	1.07 (0.97, 1.19)	1.08 (0.99, 1.17)	1.08 (1.01, 1.15)^*^	1.08 (1.04, 1.12)^***^

Sex was coded as women = 1, men = 0. Age was dichotomized at 65 years (≥65 years = 1, <65 years = 0). Educational level was categorized as ≤middle school or >middle school. Alcohol consumption and smoking status were coded as ever (yes) or never (no). Regular exercise was coded as no = 1, yes = 0. The GDS score was treated as a continuous variable. The HOMA-IR was dichotomized at the median: <1.97 (low group, reference) and ≥1.97 (high group). ^†^In women aged <65 years, all 11 ever smokers had no cognitive impairment events, resulting in complete separation. Therefore, the smoking variable was excluded from the logistic regression model for this subgroup. ^‡^*p* = 0.06; Hosmer–Lemeshow *p* < 0.0001 (total population) and *p* = 0.07 (women aged ≥ 65 years); area under the curve = 0.83 (total population) and 0.73 (women aged ≥ 65 years). ^*^p < 0.05, ^**^p < 0.01, ^***^p < 0.0001.

OR, odds ratio; CI, confidence interval; HOMA-IR, homeostatic model assessment of insulin resistance; SBP, systolic blood pressure (mm Hg); DBP, diastolic blood pressure (mm Hg); WC, waist circumference (cm); muscle mass (kg); TC, total cholesterol (mg/dL); GDS, Geriatric Depression Scale (score).

To ensure the robustness of these findings, we performed a sensitivity analysis by modeling HOMA-IR as a continuous variable. The results were consistent with our primary findings, remaining statistically significant in the total population (OR: 1.06; 95% CI: 1.01–1.12, *p* = 0.02) and among women aged ≥65 years (OR: 1.09; 95% CI: 1.02–1.16, *p* = 0.01) (Supplementary Table 7).

### 3.4 Sex-specific differences in cognitive subdomains and model validation

Following the exclusion of fasting insulin and fasting glucose to resolve multicollinearity, all retained variables in the final multivariable regression model showed acceptable variance inflation factor (VIF) values (all < 5, ranging from 1.05 to 4.80), confirming that multicollinearity was successfully resolved (Supplementary Table 8).

In the total population, mean scaled scores were the highest for memory registration (99.7) and place orientation (99.2), whereas they were the lowest for attention and calculation (67.8) and memory recall (70.7) (Supplementary Table 1). Sex-stratified analyses revealed contrasting patterns. Specifically, women in the HHG group showed significantly lower total MMSE scores (*p* < 0.0001) and poorer performance across five of the seven subdomains—excluding place orientation and memory registration—based on unadjusted *p*-values (Supplementary Table 3). To account for multiple testing across the seven subdomains, the conservative Bonferroni correction was applied. Notably, even after this rigorous adjustment, the differences among women in the HHG remained robustly significant for time orientation (adjusted *p* = 0.007), attention and calculation (adjusted *p* = 0.014), oral and written language ability (adjusted *p* < 0.0007), and visuospatial function (adjusted *p* < 0.0007). Conversely, men in the HHG scored significantly higher in total MMSE scores (*p* = 0.019); however, regarding the individual subdomains, while attention and calculation showed a baseline difference (raw *p* = 0.009), no subdomain retained statistical significance in men after the Bonferroni adjustment (all adjusted *p* > 0.05).

The multivariable model for women aged ≥65 years showed adequate calibration (*P_H_*–*_L_* = 0.07) and acceptable discrimination (AUC = 0.73), supporting the robustness of the findings. These results confirmed the reliability of the observed association between IR and cognitive impairment in the high-risk demographic.

## 4 Discussion

This cross-sectional study demonstrated a significant association between IR and cognitive impairment, with sex acting as a primary interaction factor. This association was pronounced exclusively in women aged ≥65 years. Conversely, no significant associations were observed in women aged <65 years or in men of either age group. Interaction analysis confirmed a significant sex-by-IR interaction, whereas the interaction with age was not significant. These findings suggest that sex, rather than age, may play a more prominent role in the association between insulin resistance and cognitive impairment.

Our study utilized a median HOMA-IR threshold of 1.97 to define insulin resistance. This value aligns well with the clinically validated cut-off ranges proposed by Gayoso-Diz et al. (2013), who established gender- and age-specific thresholds for metabolic risk in non-diabetic individuals within the general adult population. Given that our threshold falls within the range of these validated clinical cut-offs (e.g., 1.85 for men and 2.07 for middle-aged women), our findings regarding the association between insulin resistance and cognitive impairment are supported by prior evidence regarding metabolic health.

Unlike prior studies that reported sex-stratified associations without formal interaction testing (Kim et al., 2024), our significant sex-by-IR interaction (*p* = 0.04) provides statistical evidence that the association between IR and cognitive impairment differs meaningfully between men and women.

Our results reinforce IR as a modifiable factor for cognitive impairment, consistent with Kong et al. (2018) in older Korean adults. Furthermore, age- and sex-stratified analyses identified women aged ≥65 years as a high-risk group. This finding extends the evidence from younger cohorts; for instance, a prior study on a middle-aged sample (mean age 48.7 years) confirmed that IR is associated with decrements in global cognitive functioning and working memory (Gruber et al., 2024). Similarly, a longitudinal study with a mean age of 49.3 years reported that higher IR predicts steeper declines in verbal fluency across the total population (Ekblad et al., 2017). Furthermore, a cross-sectional analysis with a mean age of 48 years linked IR to poorer executive function, particularly in women (Schuur et al., 2010). Collectively, these studies involve data from participants in their late 40s, providing robust evidence that this specific midlife period is decisive for preventing irreversible cognitive impairment.

In contrast, our study—with a mean age of 66.6 years (range 54–86 years)—identified a far more pervasive impact through sex-stratified analysis (Supplementary Table 3). Within the women group, the high HOMA-IR group exhibited significantly lower scores in the total MMSE score, as well as in specific subdomains including time orientation, attention and calculation, oral and written language ability, and visuospatial function, compared to the low HOMA-IR group (all adjusted *p* < 0.05, Supplementary Table 3). Notably, the most pronounced deficits were observed in oral and written language ability and visuospatial function, which remained the most significantly affected domains after rigorous multiple comparison correction. The disproportionate decline in language and visuospatial functions observed in our study may be explained by the brain’s metabolic and functional architecture. Given that the human brain is composed of large-scale functional networks fueled by glucose metabolism, which account for a substantial portion of the brain’s total metabolic demand (Klug et al., 2024), high-order networks—such as those mediating language and visuospatial functions—are inherently more vulnerable to disruptions in glucose supply caused by insulin resistance. Furthermore, these domains are deeply integrated within the posterior cortical architecture, where language performance acts as a sensitive behavioral proxy for detecting underlying visuospatial processing abnormalities (Rezaii et al., 2024). Consequently, systemic insulin resistance, by compromising brain glucose availability, likely triggers a simultaneous breakdown across these functionally coupled networks, manifesting as the significant cognitive impairments observed particularly in the women in our cohort. Such associations remain significant even independently of amyloid-beta and tau lesions (Arnold et al., 2018), underscoring IR as a distinct pathogenetic contributor to cognitive impairment in older women.

Interestingly, men in the high HOMA-IR group exhibited significantly higher total MMSE scores. However, our stratified analyses suggest this is likely not a direct metabolic benefit. Supplementary Table 6 reveals that the high HOMA-IR group had significantly greater muscle mass than the low HOMA-IR group (41.7 kg vs. 40.3 kg, *p* < 0.0001). Given that muscle mass serves as a key protective factor for cognition (Supplementary Table 5) and shows significant sexual dimorphism (Supplementary Table 4), the observed cognitive advantage likely reflects residual confounding by muscle mass. This higher baseline muscle mass acts as a ‘cognitive reserve’ that masks potential cognitive impairment from metabolic dysfunction. Future research incorporating muscle quality (e.g., myosteatosis) and physical activity intensity is warranted to disentangle these complex associations.

The sex-specific association observed in our study (Table 3) may be understood within the framework of the loss of estrogen’s neuroprotective role. Notably, brain IR has been linked to impaired synaptic integrity, whereas amyloid-β and tau pathology are suggested to interfere with insulin signaling at the synapse (Kellar and Craft, 2020). These findings are consistent with proposed mechanisms involving estrogen deficiency, which may disrupt energy metabolism and render menopause a potential risk factor for IR independent of age (De Paoli et al., 2021). Furthermore, biological plausibility for our results is supported by evidence that insulin signaling modulates the molecular cascades underlying hippocampal plasticity, learning, and memory (Spinelli et al., 2019); however, our cross-sectional study does not provide direct evidence for these molecular pathways.

These findings are consistent with proposed mechanisms underlying the pronounced association between IR and cognitive impairment observed in women aged ≥65 years (Table 4). Specifically, biological plausibility for our findings is supported by previous literature suggesting that estrogen receptor beta in astrocytes modulates cognitive function (Itoh et al., 2023), and its loss during female senescence may precipitate hippocampal dysfunction. Furthermore, lower brain glucose metabolism during normal aging is predominantly observed in the frontal and temporal regions (Deery et al., 2023), which is hypothesized to synergize with IR to accelerate synaptic degradation within these vulnerable regions, although further research is required to confirm these associations in this population.

Our findings identify systemic insulin resistance as a primary determinant of cognitive impairment, with a notably heightened vulnerability in women. This sex-specific vulnerability is supported by a multi-level mechanistic framework. Recent evidence indicates that metabolic dysregulation is robustly associated with cerebral glucose hypometabolism, representing a fundamental metabolic defect in the aging brain (Jensen et al., 2025). At the molecular level, chronic insulin resistance induces metabolic stress, activating the serum response factor (SRF) and myocardin-related transcription factor-A (MRTF-A) signaling pathway; while initially an adaptive survival response, its chronic activation underlies a pathological cascade of astrocyte activation, neuroinflammation, and subsequent neurodegeneration (Jang et al., 2025). Furthermore, clinical trials such as the Arterial Stiffness, Cognition, and Equol (ACE) trial demonstrate that targeting the estrogen receptor-beta (ERβ) serves as a critical mediator in mitigating neurovascular and cognitive aging (Sekikawa et al., 2025). Collectively, these insights suggest that the post-menopausal loss of estrogen-mediated neuroprotection sensitizes the female brain to insulin-resistant neurodegeneration, emphasizing the clinical necessity of sex-specific metabolic interventions.

Beyond endogenous pathways, our study underscores the significant interplay of socio-behavioral determinants. In women aged ≥65 years, low education aligns with the cognitive reserve hypothesis (Stern, 2012), suggesting that lower education diminishes cognitive reserve against metabolic stressors. Additionally, the association between physical inactivity and cognitive impairment aligns with recent Framingham Heart Study evidence, which reported that higher levels of physical activity in both midlife and late life are associated with similar reductions in dementia risk (Marino et al., 2025). Finally, the independent contribution of depressive symptoms supports the framework of impaired cortical synaptic plasticity in late-life depression (Mirjalili et al., 2026). Collectively, these factors characterize a distinct cognitive impairment profile of older women with IR. As metabolic and hormonal shifts that predispose women to cognitive decline often accelerate during the menopausal transition, midlife represents a crucial window for intervention. Therefore, proactive metabolic management starting from midlife is essential to mitigate the heightened risk of cognitive impairment observed in women aged ≥65 years.

In evaluating the cognitive severity spectrum, mean HOMA-IR levels progressively increased from normal cognition (2.4 ± 1.7) to MCI (2.6 ± 1.8) and SCI (2.9 ± 2.9) (*p* < 0.0001, *P*-for-trend = 0.0018), with post-hoc analysis confirming significantly higher levels in impaired groups compared to the normal group. Similarly, the proportion of participants in the high HOMA-IR group (≥ 1.97) expanded across the spectrum from 48.9 to 56.9 and 54.6% (Chi-square *p* = 0.0020, *P*-for-trend = 0.0015). Although the small sample size of the SCI group (*N* = 97) limited further stratified analysis, this consistent upward trend indicates that advanced metabolic vulnerability may exacerbate the severity of cognitive impairment. Furthermore, while a uniform K-MMSE ≤ 23 threshold was used, educational level was rigorously adjusted for in all multivariate models to eliminate education-dependent screening bias, ensuring our findings are robust against educational confounding.

### 4.1 Strengths and limitations

Strengths of this study include the large, population-based KoGES Ansan-Ansung cohort, which enhances generalizability to middle-aged and older adults. Furthermore, comprehensive covariate adjustment was performed to mitigate potential confounding, allowing for the evaluation of the independent association between IR and cognitive impairment. Notably, MMSE subdomain analysis provided granular insights, revealing sex-specific deficits in language and visuospatial function exclusively among women. Importantly, a significant sex-by-IR interaction identified sex as an interaction factor. Although the age interaction was non-significant, its strong independent effect prompted sex- and age-stratified analyses to examine life-stage–specific associations. Rigorous validation further corroborated these findings, specifically, the multivariable model for women aged ≥65 years demonstrated favorable calibration and acceptable.

Nonetheless, some limitations warrant consideration. The cross-sectional design precludes causal inference between IR and cognitive impairment. Additionally, the MMSE may exhibit ceiling effects, potentially limiting its sensitivity to subtle cognitive changes. Furthermore, although rigorous statistical adjustment was applied, residual confounding from unmeasured variables cannot be entirely ruled out. Moreover, we performed multiple subgroup analyses without formal correction for multiple comparisons. Applying a Bonferroni correction, the sex-by-HOMA-IR interaction (*p* = 0.04) and the association in women aged ≥65 (*p* = 0.04) would not remain statistically significant at the corrected threshold. These findings should therefore be interpreted as exploratory and require confirmation in independent samples. Specifically, data on menopausal status, APOE genotype, diabetes duration, and the use of specific antidiabetic medications (such as metformin or insulin) were unavailable. Because these factors independently influence both metabolic profiles and cognitive trajectories, their absence may have introduced residual confounding, potentially over- or underestimating the observed sex-specific associations. Notably, the attenuated association observed in men likely reflects a selective survival bias (Mazure and Swendsen, 2016; Shaw et al., 2021). As death is a competing risk that censors the observation of cognitive decline, men who are most vulnerable to both metabolic dysregulation and cognitive impairment may be underrepresented due to earlier mortality. This survival bias poses a significant limitation in cross-sectional designs, as it potentially obscures the true association between insulin resistance and cognitive outcomes. We are currently linking our data with national mortality registries to perform a future longitudinal study utilizing competing risk models to clarify these sex-specific trajectories. Nevertheless, this does not preclude the potential for additional biological mechanisms—such as sex-specific hormonal shifts—contributing to the observed sex differences (Shaw et al., 2021).

In conclusion, this study suggests that sex may potentially interact with insulin resistance in its association with cognitive impairment, with IR as a key modifiable factor exclusively in women aged ≥65 years. However, given that the overall age interaction was not statistically significant, these age-specific findings should be interpreted with caution and considered exploratory rather than definitive. This vulnerability suggests midlife as a critical window for sex-specific metabolic management. Clinicians should prioritize IR monitoring in midlife and implement multimodal interventions addressing IR, inactivity, and depression in older women to preserve cognitive resilience. By mitigating the synergistic effects of insulin dysregulation (Kellar and Craft, 2020) and impaired cortical synaptic plasticity associated with depressive symptoms (Mirjalili et al., 2026), targeted multimodal interventions can maintain cognitive integrity and bolster resilience in older women. The link between physical inactivity and cognitive impairment further underscores the necessity of lifestyle modifications. Future longitudinal studies should elucidate the interplay between metabolic and sex-specific hormonal changes to develop personalized, life-stage-specific intervention strategies.

## Data Availability

The data analyzed in this study is subject to the following licenses/restrictions: The KoGES Ansan and Ansung cohort data, which are part of the KoGES, are publicly available at https://nih.go.kr/ and can be accessed after evaluation of the research plan by the National Institute of Health, the Korea Disease Control and Prevention Agency. Requests to access these datasets should be directed to https://www.nih.go.kr/, Email: coda@korea.kr.
