# Supplementary material for: Interaction of sex and insulin resistance with cognitive impairment in community-dwelling middle-aged and older adults
Source: Front Aging Neurosci. 2026 Oct 16;18:1889416. doi: 10.3389/fnagi.2026.1889416 (PMC13719425; doi:10.3389/fnagi.2026.1889416)
Supplement: Supplementary file 1 [file Data_Sheet_1.docx]

Supplementary Material

# 1 Supplementary Tables

**Supplementary Table 1.** Summary of the Mini-Mental State Examination domain scores in the study population

| Domain | Max points | Mean | SD | Mean scaled score |
| --- | --- | --- | --- | --- |
| Time Orientation | 5 | 4.70 | 0.8 | 94.0 |
| Place Orientation | 5 | 4.96 | 0.3 | 99.2 |
| Memory Registration | 3 | 2.99 | 0.1 | 99.7 |
| Attention and Calculation | 5 | 3.39 | 1.7 | 67.8 |
| Memory Recall | 3 | 2.12 | 0.9 | 70.7 |
| Oral and Written Language Ability | 8 | 7.52 | 0.8 | 94.0 |
| Visuospatial Function | 1 | 0.77 | 0.4 | 77.0 |
| Total MMSE Score | 30 | 26.46 | 3.4 | 88.2 |

Note: N = 3,898. Mean scaled score was calculated as (mean score/maximum points) × 100.

Abbreviations: MMSE, Mini-Mental State Examination; SD, standard deviation.

**Supplementary Table 2.** Summary statistics of continuous variables in the study population

| Variable | N | Mean | SD | Minimum | Maximum | Q1 | Median | Q3 | Range | IQR |
| --- | --- | --- | --- | --- | --- | --- | --- | --- | --- | --- |
| Age, year (continuous) | 3,898 | 66.6 | 8.2 | 54 | 86 | 60 | 65 | 73 | 32 | 13 |
| BMI, kg/m^2^ | 3,834 | 24.7 | 3.2 | 14.1 | 38.2 | 22.5 | 24.6 | 26.7 | 24.1 | 4.2 |
| SBP, mm Hg | 3,881 | 125.1 | 17.2 | 80 | 220 | 112 | 124 | 136 | 140 | 24 |
| DBP, mm Hg | 3,881 | 78.1 | 9.9 | 48 | 118 | 70 | 78 | 84 | 70 | 14 |
| WC, cm | 3,898 | 86.0 | 8.7 | 56.3 | 125.0 | 80.3 | 86.2 | 91.5 | 68.7 | 11.2 |
| Muscle mass, kg | 3,834 | 41.0 | 8.2 | 21.2 | 67.3 | 34.5 | 39.3 | 47.2 | 46.1 | 12.7 |
| TC, mg/dL | 3,897 | 186.0 | 37.3 | 66 | 362 | 161 | 185 | 211 | 296 | 50 |
| TG, mg/dL | 3,895 | 136.3 | 86.7 | 32 | 1,157 | 87 | 116 | 159 | 1,125 | 72 |
| HDL-C, mg/dL | 3,897 | 45.9 | 11.8 | 14 | 104 | 37 | 45 | 53 | 90 | 16 |
| Vitamin D, ng/mL | 3,673 | 21.0 | 10.2 | 4.0 | 150.0 | 13.6 | 19.4 | 26.5 | 146.0 | 12.9 |
| hsCRP, mg/L | 3,897 | 1.78 | 5.34 | 0.01 | 151.81 | 0.40 | 0.71 | 1.46 | 151.80 | 1.06 |
| Fasting glucose, mg/dL | 3,898 | 99.6 | 26.1 | 39 | 409 | 86 | 93 | 103 | 370 | 17 |
| Fasting insulin, µIU/mL | 3,898 | 9.5 | 5.2 | 1.0 | 75.5 | 6.4 | 8.3 | 11.1 | 74.5 | 4.7 |
| HOMA-IR (continuous) | 3,898 | 2.40 | 1.80 | 0.14 | 36.17 | 1.46 | 1.97 | 2.75 | 36.02 | 1.30 |
| GDS score (continuous) | 3,891 | 2.08 | 3.1 | 0 | 15 | 0 | 1 | 3 | 15 | 3 |
| MMSE score (continuous) | 3,898 | 26.46 | 3.4 | 5 | 30 | 25 | 27 | 29 | 25 | 4 |

Abbreviations: BMI, body mass index; SBP, systolic blood pressure; DBP, diastolic blood pressure; WC, waist circumference; TC, total cholesterol; TG, triglycerides; HDL-C, high-density lipoprotein cholesterol; hs-CRP, high-sensitivity C-reactive protein; GDS, Geriatric Depression Scale; MMSE, Mini-Mental State Examination; HOMA-IR, homeostatic model assessment of insulin resistance; Q1, first quartile; Q3, third quartile; SD, standard deviation; IQR, interquartile range.

**Supplementary Table 3.** Mini-Mental State Examination domain scores by insulin resistance group and sex

| Domain | Max score | Men (n = 1,735) | | | | | Women (n = 2,163) | | | | |
| --- | --- | --- | --- | --- | --- | --- | --- | --- | --- | --- | --- |
|  |  | LHG  (<1.97),  n = 884  (51.0%) | | HHG (≥1.97),  n = 851  (49.0%) | Raw  *P-*value | Bonferroni Adjusted *P*-value | | LHG  (<1.97),  n = 1,058  (48.9%) | HHG  (≥1.97),  n = 1,105 (51.1%) | Raw  *P-*value | Bonferroni Adjusted P-value |
| Time Orientation | 5 | 4.76 ± 0.6 | | 4.77 ± 0.6 | 0.718 | 1.000 | | 4.70 ± 0.7 | 4.58 ± 0.9 | 0.001 | 0.007 |
| Place Orientation | 5 | 4.97 ± 0.2 | | 4.98 ± 0.1 | 0.069 | 0.483 | | 4.94 ± 0.3 | 4.94 ± 0.3 | 0.679 | 1.000 |
| Memory Registration | 3 | 2.99 ± 0.1 | | 3.00 ± 0.1 | 0.565 | 1.000 | | 2.99 ± 0.1 | 2.99 ± 0.1 | 0.249 | 1.000 |
| Attention and Calculation | 5 | 3.72 ± 1.6 | | 3.91 ± 1.5 | 0.009 | 0.063 | | 3.18 ± 1.8 | 2.94 ± 1.8 | 0.002 | 0.014 |
| Memory Recall | 3 | 2.09 ± 1.0 | | 2.14 ± 0.9 | 0.214 | 1.000 | | 2.18 ± 0.9 | 2.09 ± 1.0 | 0.018 | 0.126 |
| Oral and Written Language Ability | 8 | 7.64 ± 0.7 | | 7.67 ± 0.6 | 0.361 | 1.000 | | 7.50 ± 0.9 | 7.34 ± 1.0 | <0.0001 | <0.0007 |
| Visuospatial Function | 1 | 0.85 ± 0.4 | | 0.85 ± 0.4 | 0.941 | 1.000 | | 0.75 ± 0.4 | 0.66 ± 0.5 | <0.0001 | <0.0007 |
| Total MMSE Score | 30 | 27.01 ± 2.8 | 27.32 ± 2.6 | | 0.019 | - | | 26.25 ± 3.5 | 25.55 ± 3.8 | <0.0001 | - |

Continuous variables are presented as mean ± SD. *P*-values were calculated using Student’s *t*-test for continuous variables. HOMA-IR was dichotomized at the median value: <1.97 (low HOMA-IR group, LHG) and ≥1.97 (high HOMA-IR group, HHG). Adjusted *P*-values were calculated using the Bonferroni correction method by multiplying the raw *P*-values by 7, representing the number of MMSE subdomains. Adjusted *P*-values exceeding 1.000 are designated as 1.000. Total MMSE score was excluded from the subdomain multiplicity adjustment.

Abbreviations: HHG, high HOMA-IR group; HOMA-IR, homeostatic model assessment of insulin resistance; LHG, low HOMA-IR group; MMSE, Mini-Mental State Examination; SD, standard deviation.

**Supplementary Table 4.** Clinical characteristics and cognitive function stratified by sex

| Variable | Category | Men  (n = 1,735) | Women  (n = 2,163) | *P-*value |
| --- | --- | --- | --- | --- |
| Age, year (continuous) | - | 66.0 ± 7.9 | 67.2 ± 8.4 | <0.0001 |
| Age, year (categorical) | <65 | 905 (46.7) | 1,034 (53.3) | 0.007 |
|  | ≥65 | 830 (42.4) | 1,129 (57.6) |  |
| Education | ≤Middle school | 726 (32.4) | 1,513 (67.6) | <0.0001 |
|  | ≥High school | 999 (61.3) | 630 (38.7) |  |
| Alcohol consumption | Never drinker | 456 (20.1) | 1,814 (79.9) | <0.0001 |
|  | Past drinker | 224 (89.2) | 27 (10.8) |  |
|  | Current drinker | 1,054 (76.6) | 322 (23.4) |  |
| Smoking | Never smoker | 1,304 (49.9) | 1,312 (50.1) | 0.771 |
|  | Past smoker | 440 (49.1) | 456 (50.9) |  |
|  | Current smoker | 198 (51.3) | 188 (48.7) |  |
| Regular exercise | No | 1,113 (42.2) | 1,525 (57.8) | <0.0001 |
|  | Yes | 622 (49.4) | 638 (50.6) |  |
| BMI, kg/m^2^ | - | 24.5 ± 3.0 | 24.9 ± 3.4 | 0.001 |
| SBP, mm Hg | - | 124.9 ± 15.7 | 125.2 ± 18.4 | 0.474 |
| DBP, mm Hg | - | 79.3 ± 10.0 | 77.1 ± 9.8 | <0.0001 |
| WC, cm | - | 87.2 ± 7.9 | 85.1 ± 9.1 | <0.0001 |
| Muscle mass, kg | - | 48.1 ± 6.1 | 35.3 ± 4.1 | <0.0001 |
| TC, mg/dL | - | 180.0 ± 36.0 | 190.7 ± 37.6 | <0.0001 |
| TG, mg/dL | - | 142.9 ± 99.9 | 131.0 ± 74.1 | <0.0001 |
| HDL-C, mg/dL | - | 43.4 ± 11.4 | 47.9 ± 11.7 | <0.0001 |
| Vitamin D, ng/mL | - | 21.2 ± 9.0 | 20.8 ± 11.2 | 0.190 |
| hsCRP, mg/L | - | 2.0 ± 5.9 | 1.6 ± 4.8 | 0.087 |
| Fasting glucose, mg/dL | - | 102.0 ± 25.5 | 97.7 ± 26.4 | <0.0001 |
| Fasting insulin, µIU/mL | - | 9.0 ± 4.6 | 9.9 ± 5.5 | <0.0001 |
| HOMA-IR (continuous) | - | 2.3 ± 1.5 | 2.5 ± 2.0 | 0.005 |
| HOMA-IR (categorical) | ≥1.97 (high group) | 851 (43.5) | 1105 (56.5) | 0.206 |
|  | <1.97 (low group) | 884 (45.5) | 1058 (54.5) |  |
| GDS score (continuous) | - | 1.6 ± 2.7 | 2.4 ± 3.3 | <0.0001 |
| GDS score (categorical) | ≥6 (depressive symptoms) | 161 (33.1) | 326 (66.9) | <0.0001 |
|  | <6 (normal) | 1,571 (46.2) | 1,833 (53.8) |  |
| MMSE score (continuous) | - | 27.2 ± 2.7 | 25.9 ± 3.7 | <0.0001 |
| MMSE score (categorical) | ≤23 (cognitive impairment) | 164 (25.8) | 471 (74.2) | <0.0001 |
|  | ≥24 (normal cognition) | 1,571 (48.2) | 1,692 (51.8) |  |

HOMA-IR was dichotomized at the median (≥1.97 = high group; <1.97 = low group). GDS score was categorized using a cutoff of ≥6 to indicate depressive symptoms. An MMSE score ≤23 was used to define cognitive impairment. Educational level was categorized as ≤middle school or ≥high school. Alcohol consumption and smoking status were classified as never, past, or current users. Regular exercise was coded as yes or no. Continuous variables are presented as mean ± standard deviation, while categorical variables are expressed as number (%). Sex-specific differences were assessed using Student’s t-test and the chi-squared test for continuous and categorical variables, respectively.

Abbreviations: BMI, body mass index; DBP, diastolic blood pressure; GDS, Geriatric Depression Scale; HDL-C, high-density lipoprotein cholesterol; HOMA-IR, homeostatic model assessment of insulin resistance; hsCRP, high-sensitivity C-reactive protein; MMSE, Mini-Mental State Examination; SBP, systolic blood pressure; TC, total cholesterol; TG, triglycerides; WC, waist circumference.

**Supplementary Table 5.** Association between participant characteristics and cognitive impairment

| Variable | Category | Cognitive impairment | | Normal  cognition | | *P*-value |
| --- | --- | --- | --- | --- | --- | --- |
|  |  | ≤23,  n = 635  (16.3%) | | ≥24,  n = 3,263 (83.7%) | |  |
| Sex | Men | | 164 (9.5) | | 1,571 (90.5) | <0.0001 |
|  | Women | | 471 (21.8) | | 1,692 (78.2) |  |
| Age, year (continuous) | - | | 74.1 ± 7.6 | | 65.2 ± 7.5 | <0.0001 |
| Age, year (categorical) | <65 | | 97 (5.0) | | 1,842 (95.0) | <0.0001 |
|  | ≥65 | | 538 (27.5) | | 1,421 (72.5) |  |
| Education | ≤Middle school | | 573 (25.6) | | 1,666 (74.4) | <0.0001 |
|  | ≥High school | | 50 (3.1) | | 1,579 (96.9) |  |
| Alcohol consumption | Never drinker | | 458 (20.2) | | 1,812 (79.8) | <0.0001 |
|  | Past drinker | | 38 (15.1) | | 213 (84.9) |  |
|  | Current drinker | | 139 (10.1) | | 1,237 (89.9) |  |
| Smoking | Never smoker | | 517 (19.8) | | 2,099 (80.2) | <0.0001 |
|  | Past smoker | | 75 (8.4) | | 821 (91.6) |  |
|  | Current smoker | | 43 (11.1) | | 343 (88.9) |  |
| Regular exercise | No | | 532 (20.2) | | 2,106 (79.8) | <0.0001 |
|  | Yes | | 103 (8.2) | | 1,157 (91.8) |  |
| BMI, kg/m^2^ | - | | 24.6 ± 3.7 | | 24.7 ± 3.1 | 0.418 |
| SBP, mm Hg | - | | 130.7 ± 19.1 | | 124.0 ± 16.6 | <0.0001 |
| DBP, mm Hg | - | | 77.4 ± 10.3 | | 78.2 ± 9.9 | 0.063 |
| WC, cm | - | | 86.9 ± 9.3 | | 85.8 ± 8.5 | 0.006 |
| Muscle mass, kg | - | | 36.0 ± 6.8 | | 41.9 ± 8.1 | <0.0001 |
| TC, mg/dL | - | | 180.1 ± 35.6 | | 187.1 ± 37.5 | <0.0001 |
| TG, mg/dL | - | | 136.6 ± 89.1 | | 136.3 ± 86.2 | 0.931 |
| HDL-C, mg/dL | - | | 45.5 ± 11.7 | | 45.9 ± 11.8 | 0.400 |
| Vitamin D, ng/mL | - | | 20.9 ± 10.9 | | 21.0 ± 10.0 | 0.814 |
| hsCRP, mg/L | - | | 1.9 ± 4.6 | | 1.8 ± 5.5 | 0.397 |
| Fasting glucose, mg/dL | - | | 102.2 ± 31.4 | | 99.1 ± 24.9 | 0.017 |
| Fasting insulin, µIU/mL | - | | 10.3 ± 5.9 | | 9.3 ± 5.0 | 0.001 |
| HOMA-IR (continuous) | - | | 2.7 ± 2.0 | | 2.4 ± 1.7 | 0.001 |
| HOMA-IR (categorical) | ≥1.97 (high group) | | 359 (18.4) | | 1,597 (81.6) | 0.001 |
|  | <1.97 (low group) | | 276 (14.2) | | 1,666 (85.8) |  |
| GDS score (continuous) | - | | 3.3 ± 3.9 | | 1.8 ± 2.8 | <0.0001 |
| GDS score (categorical) | ≥6 (depressive symptoms) | | 152 (31.2) | | 335 (68.8) | <0.0001 |
|  | <6 (normal) | | 481 (14.1) | | 2,923 (85.9) |  |
| MMSE score (continuous) | - | | 20.4 ± 2.8 | | 27.6 ± 1.8 | <0.0001 |

HOMA-IR was dichotomized at the median (≥1.97 = high group; <1.97 = low group). GDS score was categorized using a cutoff of ≥6 to indicate depressive symptoms. Educational level was categorized as ≤ middle school or ≥ high school. Alcohol consumption and smoking status were classified as never, past, or current users. Regular exercise was coded as yes or no. Continuous variables are presented as mean ± standard deviation, while categorical variables are expressed as number (%). Sex-specific differences were assessed using Student’s t-test and the chi-squared test for continuous and categorical variables, respectively.

Abbreviations: BMI, body mass index; DBP, diastolic blood pressure; GDS, Geriatric Depression Scale; HDL-C, high-density lipoprotein cholesterol; HOMA-IR, homeostatic model assessment of insulin resistance; hsCRP, high-sensitivity C-reactive protein; MMSE, Mini-Mental State Examination; SBP, systolic blood pressure; TC, total cholesterol; TG, triglycerides; WC, waist circumference.

**Supplementary Table 6.** Association between participant characteristics and insulin resistance group

| Variable | Category | Low  HOMA-IR  (LHG <1.97) | High  HOMA-IR  (HHG ≥1.97) | *P*-value |
| --- | --- | --- | --- | --- |
|  |  | n = 1,942 (49.8%) | n = 1,956  (50.2%) |  |
| Sex | Men | 884 (51.0) | 851 (49.0) | 0.206 |
|  | Women | 1,058 (48.9) | 1,105 (51.1) |  |
| Age, year (continuous) | - | 66.3 ± 8.4 | 67.0 ± 8.1 | 0.005 |
| Age, year (categorical) | <65 | 1,022 (52.7) | 917 (47.3) | 0.001 |
|  | ≥65 | 920 (47.0) | 1,039 (53.0) |  |
| Education | ≤Middle school | 1,047 (46.8) | 1,192 (53.2) | <0.0001 |
|  | ≥High school | 884 (54.3) | 745 (45.7) |  |
| Alcohol consumption | Never drinker | 1,125 (49.6) | 1,145 (50.4) | 0.046 |
|  | Past drinker | 108 (43.0) | 143 (57.0) |  |
|  | Current drinker | 708 (51.5) | 668 (48.5) |  |
| Smoking | Never smoker | 1,304 (49.9) | 1,312 (50.1) | 0.771 |
|  | Past smoker | 440 (49.1) | 456 (50.9) |  |
|  | Current smoker | 198 (51.3) | 188 (48.7) |  |
| Regular exercise | No | 1,266 (48.0) | 1,372 (52.0) | 0.001 |
|  | Yes | 676 (53.7) | 584 (46.3) |  |
| BMI, kg/m^2^ | - | 23.4 ± 2.8 | 26.0 ± 3.1 | <0.0001 |
| SBP, mm Hg | - | 123.0 ± 17.3 | 127.1 ± 17.0 | <0.0001 |
| DBP, mm Hg | - | 77.1 ± 10.1 | 79.0 ± 9.7 | <0.0001 |
| WC, cm | - | 82.5 ± 8.1 | 89.5 ± 7.8 | <0.0001 |
| Muscle mass, kg | - | 40.3 ± 8.0 | 41.7 ± 8.3 | <0.0001 |
| TC, mg/dL | - | 190.3 ± 36.8 | 181.7 ± 37.3 | <0.0001 |
| TG, mg/dL | - | 117.2 ± 67.6 | 155.4 ± 98.6 | <0.0001 |
| HDL-C, mg/dL | - | 48.7 ± 11.9 | 43.0 ± 10.9 | <0.0001 |
| Vitamin D, ng/mL | - | 22.1 ± 10.4 | 19.9 ± 9.8 | <0.0001 |
| hsCRP, mg/L | - | 1.6 ± 4.6 | 2.0 ± 5.9 | 0.013 |
| Fasting glucose, mg/dL | - | 89.4 ± 11.4 | 109.7 ± 32.0 | <0.0001 |
| Fasting insulin, µIU/mL | - | 6.5 ± 1.5 | 12.5 ± 5.7 | <0.0001 |
| HOMA-IR (continuous) | - | 1.4 ± 0.3 | 3.4 ± 2.1 | <0.0001 |
| GDS score (continuous) | - | 2.1 ± 3.1 | 2.1 ± 3.1 | 0.836 |
| GDS score (categorical) | ≥6 (depressive symptoms) | 1,701 (50.0) | 1,703 (50.0) | 0.590 |
|  | <6 (normal) | 237 (48.7) | 250 (51.3) |  |
| MMSE score (categorical) | ≤23 (cognitive impairment) | 276 (43.5) | 359 (56.5) | 0.001 |
|  | ≥24 (normal cognition) | 1,666 (51.1) | 1,597 (48.9) |  |

HOMA-IR was dichotomized at the median value: <1.97 (low HOMA-IR group, LHG) and ≥1.97 (high HOMA-IR group, HHG). Continuous variables are presented as mean ± standard deviation; categorical variables are expressed as No. (%).

*P* values were calculated using Student’s t-test and the χ² test for continuous and categorical variables, respectively.

Abbreviations: BMI, body mass index; DBP, diastolic blood pressure; GDS, Geriatric Depression Scale; HDL-C, high-density lipoprotein cholesterol; HOMA-IR, homeostatic model assessment of insulin resistance; hs-CRP, high-sensitivity C-reactive protein; MMSE, Mini-Mental State Examination; SBP, systolic blood pressure; TC, total cholesterol; TG, triglycerides; WC, waist circumference.

**Supplementary Table 7.** Sensitivity analysis: Multivariable logistic regression analysis for factors associated with cognitive impairment treating HOMA-IR as a continuous variable

| Variables |  | Cognitive impairment, OR (95% CI) | | | | | | |
| --- | --- | --- | --- | --- | --- | --- | --- | --- |
|  | Total  population | | Age <65 years (n = 1,939, 49.7%) | | Age ≥65 years (n = 1,959, 50.3%) | | | |
|  |  |  | Men  (n = 905,  46.7%) | Women  (n = 1,034,  53.3%) | | Men  (n = 830,  42.4%) | Women  (n = 1,129,  57.6%) |  |
| HOMA-IR (continuous) | 1.06  (1.01, 1.12)* | | 0.89  (0.66, 1.20) | 1.09  (0.95, 1.25) | | 1.00  (0.84, 1.19) | 1.09  (1.02, 1.16)* |  |
| Sex (women) | 0.48  (0.30, 0.75)** | | - | - | | - | - |  |
| Age (≥65 years) | 2.91  (2.21, 3.83)*** | | - | - | | - | - |  |
| Education  (≤middle school) | 4.25  (3.07, 5.87)*** | | 3.38  (1.74, 6.58)** | 13.34  (4.00, 44.55)*** | | 2.30  (1.38, 3.83)** | 9.85  (4.19, 22.12)*** |  |
| Alcohol consumption (ever) | 1.11  (0.85, 1.45) | | 0.67  (0.33, 1.38) | 1.01  (0.49, 2.08) | | 1.61  (0.97, 2.68) | 1.14  (0.74, 1.76) |  |
| Smoking (ever) | 0.76  (0.52, 1.11) | | 0.56  (0.27, 1.15) | -† | | 0.74  (0.46, 1.20) | 1.08  (0.33, 3.53) |  |
| Regular exercise (no) | 1.54  (1.19, 1.98)** | | 2.24  (1.03, 4.89)* | 0.89  (0.47, 1.70) | | 1.48  (0.85, 2.57) | 1.76  (1.23, 2.52)** |  |
| SBP, mm Hg | 1.01  (1.00, 1.02)** | | 1.04  (1.01, 1.07)* | 1.02  (1.00, 1.05) | | 1.01  (0.99, 1.02) | 1.01  (1.00, 1.02) |  |
| DBP, mm Hg | 0.99  (0.98, 1.01) | | 0.96  (0.91, 1.00) | 0.96  (0.92, 1.01) | | 0.99  (0.97, 1.02) | 1.00  (0.99, 1.02) |  |
| WC, cm | 1.02  (1.01, 1.03)** | | 1.02  (0.96, 1.08) | 1.03  (0.99, 1.07) | | 1.02  (0.99, 1.06) | 1.02  (1.00, 1.04) |  |
| Muscle mass, kg | 0.90  (0.87, 0.92)*** | | 0.97  (0.90, 1.04) | 0.94  (0.86, 1.03) | | 0.88  (0.84, 0.93)*** | 0.86  (0.83, 0.90)*** |  |
| TC, mg/dL | 1.00  (1.00, 1.00) | | 1.00  (0.99, 1.01) | 1.00  (0.99, 1.01) | | 1.00  (0.99, 1.01) | 1.00  (1.00, 1.00) |  |
| GDS score  (continuous) | 1.08  (1.05, 1.11)*** | | 1.06  (0.96, 1.18) | 1.07  (0.99, 1.16) | | 1.08  (1.01, 1.15)* | 1.08  (1.04, 1.12)*** |  |

Values are presented as odds ratio (95% confidence interval). Sex was coded as women = 1, men = 0. Age was dichotomized at 65 years (≥65 years = 1, <65 years = 0). Educational level was categorized as ≤middle school or >middle school. Alcohol consumption and smoking status were coded as ever (yes) or never (no). Regular exercise was coded as no = 1, yes = 0. All continuous variables (including HOMA-IR, SBP, DBP, waist circumference, muscle mass, total cholesterol, and GDS score) were modeled in their original continuous forms. †In women aged <65 years, all 11 ever smokers had no cognitive impairment events, resulting in complete separation. Therefore, the smoking variable was excluded from the logistic regression model for this subgroup. Hosmer-Lemeshow goodness-of-fit test: *p* < 0.0001 for the total population and *p* = 0.69 for women aged 65 years or older. Area under the curve (AUC): 0.83 for the total population and 0.73 for women aged 65 years or older. **p* < 0.05, ***p* < 0.01, ****p* < 0.0001.

Abbreviations: OR, odds ratio; CI, confidence interval; HOMA-IR, homeostatic model assessment of insulin resistance; SBP, systolic blood pressure; DBP, diastolic blood pressure; WC, waist circumference; TC, total cholesterol; GDS, Geriatric Depression Scale.

**Supplementary Table 8.** Variance inflation factor (VIF) and parameter estimates for covariates in

the final multivariable regression model

| Variables | Parameter Estimate | Standard Error | *P-*value | Variance Inflation (VIF) |
| --- | --- | --- | --- | --- |
| **Continuous variables** |  |  |  |  |
| HOMA-IR | -0.073 | 0.029 | 0.011 | 1.18 |
| SBP, mm Hg | -0.011 | 0.004 | 0.003 | 1.90 |
| DBP, mm Hg | 0.008 | 0.006 | 0.239 | 1.84 |
| WC, cm | -0.018 | 0.007 | 0.011 | 1.67 |
| Muscle mass, kg | 1.102 | 0.012 | < 0.0001 | 4.28 |
| TC, mg/dL | 0.002 | 0.001 | 0.149 | 1.09 |
| GDS score | -0.135 | 0.016 | < 0.0001 | 1.05 |
| **Categorical variables** |  |  |  |  |
| Sex (women) | 0.783 | 0.208 | 0.0002 | 4.80 |
| Age (≥65 years) | -1.031 | 0.117 | < 0.0001 | 1.55 |
| Education (≤middle school) | -1.482 | 0.112 | < 0.0001 | 1.37 |
| Alcohol consumption (ever) | 0.020 | 0.120 | 0.866 | 1.59 |
| Smoking (ever) | 0.164 | 0.156 | 0.295 | 2.44 |
| Regular exercise (no) | -0.374 | 0.105 | 0.0004 | 1.09 |

Values are presented as parameter estimates, standard errors, *P*-values, and variance inflation factors (VIF). The final multivariable linear regression model includes all retained covariates after the exclusion of fasting insulin and fasting glucose to resolve multicollinearity. The overall regression model was statistically significant (F = 95.67, Model *P* < 0.0001, R^2^ = 0.248, Adjusted R^2^ = 0.245). Abbreviations: HOMA-IR, homeostasis model assessment of insulin resistance; SBP, systolic blood pressure; DBP, diastolic blood pressure; WC, waist circumference; TC, total cholesterol; GDS, Geriatric Depression Scale.
